# Supplementary material for: Complexome profiling of the Chlamydomonas psb28 mutant reveals TEF5 as an early PSII assembly factor
Source: Plant Cell. 2025 Mar 17;37(6):koaf055. doi: 10.1093/plcell/koaf055 (PMC12164590; doi:10.1093/plcell/koaf055)
Supplement: koaf055_Supplementary_Data [file koaf055_supplementary_data.zip › psb28_interactive.zip!/plots/intensity/qualityPlots/peptides/NUOB10_Cre12.g555150.t1.2.html]

### Cre12.g555150.t1.2

Trivial name: NUOB10  
SeqCoverage [median]: 50.30  
Mass: 17.88 kDa  
  
 

| Sequence | MissedCleavage | Mass [Da] | wtIntensity | muIntensity | Score |
| --- | --- | --- | --- | --- | --- |
| DVGFIYHNNR | 0 | 1233.59 | 13863.87 | 10830.48 | 95.50 |
| ELQQQAAELEIENPFPAPAK | 0 | 2222.12 | 3555.02 | 11799.71 | 102.43 |
| LRELQQQAAELEIENPFPAPAK | 1 | 2491.31 | 5610.85 | 3342.16 | 83.39 |
| NLTGEAGK | 0 | 788.40 | 1766.17 | 0.00 | 69.83 |
| NLTGEAGKR | 1 | 944.50 | 3560.73 | 1539.46 | 101.72 |
| QSFHDKVNDVFK | 1 | 1462.72 | 5253.91 | 0.00 | 41.40 |
| SASVEER | 0 | 776.37 | 3716.30 | 3608.15 | 127.84 |
| VASNVDR | 0 | 759.39 | 2442.00 | 4317.63 | 126.28 |
| VASNVDRGPLAR | 1 | 1253.68 | 22347.10 | 36806.26 | 132.32 |
| YINVEK | 0 | 764.41 | 790.85 | 1870.77 | 94.16 |
|  |  |  | 62906.8 | 74114.6 |  |
